# The role of cytokines in ovarian cancer drug resistance

**DOI:** 10.1002/ijc.70099

**Published:** 2025-09-19

**Authors:** Lu Wang, Yunxia Zang, Rebeka Dejenie, Junyuan Bing, Shixia Dong, Yanfei Zhang, Yi Zhu, Jingjing Li

**Affiliations:** ^1^ School of Clinical Medicine Shandong Second Medical University Weifang China; ^2^ Jinming Yu Academician Workstation of Oncology Affiliated Hospital of Shandong Second Medical University Weifang China; ^3^ Affiliated Hospital of Shandong Second Medical University, School of Clinical Medicine Shandong Second Medical University Weifang China; ^4^ University of Chicago Medical Center Chicago Illinois USA; ^5^ Department of Orthopaedics The First Affiliated Hospital of Soochow University Suzhou China; ^6^ Orthopaedic Institute, Medical College Soochow University Suzhou China

**Keywords:** cytokine, drug resistance, IL6, ovarian cancer, tumor microenvironment

## Abstract

Ovarian cancer is the leading cause of death among women diagnosed with female reproductive system cancers. While significant advances have been made in treating various types of cancer, progress in ovarian cancer treatment over the past 20 years has been minimal, and the treatment course of ovarian cancer is not linear. Although many patients initially respond to treatment and may experience tumor regression, 80% of patients relapse after one or multiple chemotherapy sessions. Cytokines play a crucial role in driving tumor progression, relapse, and drug resistance in ovarian cancer. The knowledge of ovarian cancer drug resistance and the function of cytokines in the development of tumor resistance is currently the subject of numerous relevant studies; meanwhile, the specific molecular pathways are being unearthed. Herein, we summarize the recent advancements in cytokine‐associated ovarian cancer drug resistance. In addition, we also present the cytokine targeting drugs in ameliorating ovarian cancer drug resistance. As multi‐effect factors, cytokines can induce multiple pathways to promote tumor progression and overcome extremely complex and heterogeneous living environments. From the perspective of various paths of cytokine function, the emergence of multi‐target drugs might be more promising for conquering ovarian cancer chemotherapy drug resistance.

## Graphical abstract

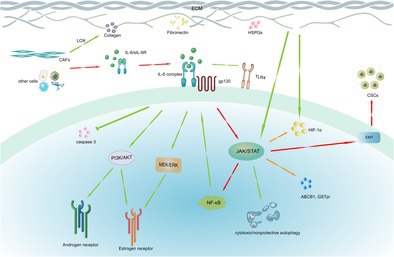

**Abbreviations**
AR: androgen receptor
CAFs: tumor‐associated fibroblasts
CSCs: cancer stem cells
CSF‐1: colony‐stimulating factor
ECM: extracellular matrix
EMT: epithelial‐to‐mesenchymal transition
FAK: focal adhesion kinase
FGF: fibroblast growth factor
FGFR: fibroblast growth factor receptor
GM‐CSF: granulocyte‐macrophage colony‐stimulating factor
IFNε: interferon‐ε
IL‐4: interleukin‐4
IL‐6: Interleukin‐6
IL‐6R: interleukin‐6 receptor
IL‐8: interleukin‐8
JAK2: Janus kinase 2
LIF: leukemia inhibitory factor
LOX: lysyl oxidase
MCP‐1: monocyte chemoattractant protein‐1
OSM: oncostatin M
PI3K/AKT: phosphoinositide 3‐kinase/protein kinase B
STAT3: signal transducer and activator of transcription 3
TGF‐β: transforming growth factor‐β
TLR4: toll‐like receptor 4
TME: tumor microenvironment
TNF: tumor necrosis factor
TNFAIP8: tumor necrosis factor α‐induced protein 8
VEGF: vascular endothelial growth factor

## 1 INTRODUCTION

Ovarian cancer is the leading cause of death among women diagnosed with female reproductive system cancers. 90% of ovarian cancers originate from epithelial cells, of which 70% are high‐grade serous carcinomas with potential intra‐abdominal invasion and metastasis.^1^ So far, platinum‐based chemotherapy is still the first‐line choice for patients with ovarian cancer. Platinum‐based drugs lead to cancer cell apoptosis by forming DNA adducts to impede DNA replication.^2^ Paclitaxel, commonly used in combination with platinum‐based drugs for ovarian cancer chemotherapy, exerts its effects by binding to β‐tubulins and inhibiting microtubule depolymerization, thereby blocking cell cycle progression.^3^ Poly‐ADP ribose polymerase inhibitors are also widely utilized as maintenance therapy for advanced ovarian cancer, providing a significant progression‐free survival benefit for patients.^4^

In the last 20 years, there has been great progress in the treatment of multiple oncologic diseases, but minimal progress in the treatment of ovarian cancer. Although many patients initially respond to treatment and may experience tumor regression, 80% of patients relapse after one or multiple chemotherapy sessions.^5^ Among them, around 75% of cases relapse into a drug‐resistant status.^6^ The mechanisms of drug resistance are rather complex and can include, but are not limited to, dysfunction of influx and efflux transporters, tubulin isotype compensation, metabolic reprogramming, Phosphoinositide 3‐kinase/protein kinase B (PI3K/AKT) pathway hyperactivation, and cisplatin‐induced autophagy.^3^, ^7^ Additionally, the role of cytokines in drug resistance is also widely investigated. Cytokines, including IL‐6, IL‐8, TGF‐β, VEGF, and EGFR et al., are secreted by multiple immune cells,^8^ leading to a complex tumor microenvironment by influencing cell–cell interactions, cell growth, and cell differentiation.^9^

While numerous studies have investigated ovarian cancer drug resistance and the role of cytokines in tumor resistance, the specific molecular pathways involved are also being unearthed. Herein, we summarize the recent advancements in cytokine‐associated ovarian cancer drug resistance. In addition, we also present the cytokine‐targeting drugs in ameliorating ovarian cancer drug resistance.

## 2 THE FAMILY OF IL‐6‐TYPE CYTOKINES

The Interleukin‐6 family of cytokines is comprised of IL‐6, IL‐11, IL‐31, Cardiotrophin‐1, Ciliary neurotrophic factor, Cardiotropic‐like cytokine, Granulocyte‐colony stimulating factor, Leptin, Leukemia inhibitory factor (LIF), Neuropoietic, and Oncostatin M. Gp130 is also a common signal transducing component for the functional receptor complexes of the IL‐6‐type‐cytokines.^10^

The IL‐6‐type cytokines have multiple roles in tumor progression. For one, IL‐6 signaling can reprogram the T cell immune response, switching it from an inhibiting state to a responsive state against tumors.^11^ Additionally, IL‐6 promotes tumor cell proliferation, survival, and angiogenesis, as well as evasion of immune surveillance to build a favorable environment for tumorigenesis. In patients with ovarian cancer, elevated levels of IL‐6 are associated with poor survival and chemoresistance.^12^ As a pleiotropic cytokine, IL‐6 performs multiple functions to boost the adaptability of cancer cells to conquer an unfavorable environment. Furthermore, IL‐6 can be considered a potential biomarker to distinguish malignant and non‐malignant lesions in ovarian tissue. Overall, IL‐6 can serve as a potential predictor of chemoresistance in ovarian cancer and may assist in identifying its occurrence.^13^

### 2.1 IL‐6/IL‐6R/gp130 complex

IL‐6 binds to interleukin‐6 receptor (IL‐6R) followed by homodimerization and recruitment of gp130. The hexameric IL‐6/IL‐6R/gp130 complex initiates the downstream signal transduction pathways, such as signal transducer and activator of transcription 3 (STAT3) signaling pathway.^14^ IL‐6 expresses signals via gp130 interacting with either membrane‐bound IL‐6R or the soluble IL‐6R, which is termed classic signaling and trans‐signaling, respectively.^15^ Ubiquitously expressed gp130 mediates multiple essential biological processes. It is a potential and promising way to alter chemoresistance by interrupting the activity of gp130. According to the biological functions of IL‐6, several antibody drugs are ingeniously designed and developed. IL‐6/IL‐6R/gp130 targeting antibodies have demonstrated beneficial outcomes in preclinical studies and clinical trials, such as in Bazedoxifene, SC144.^16^, ^17^

### 2.2 IL‐6 complex promotes multidrug resistance

A variety of tumor progression‐associated proteins are induced by IL‐6 in ovarian cancer. Aside from its benefit to cancer cell survival and angiogenesis, IL‐6 also induces resistance to Cisplatin and Paclitaxel. As stated previously, high expression of IL‐6 positively correlates with ovarian cancer drug resistance. Wang et al.^13^ report that ovarian cancer cells, SKOV3, become more sensitive to Cisplatin and Paclitaxel when the IL‐6 expression level declines. Mechanistically, IL‐6 not only inhibits the proteolytic activation of Caspase‐3 but also upregulates the expression of drug resistance‐related genes, including *ABCB1* and *GSTpi*.^13^

### 2.3 IL‐6 increases the expression of HIF‐1 to aggravate drug resistance

SC144, an inhibitor of Gp‐130, upregulates the transcription of hypoxia‐inducible factor antisense. This occurs in alignment with the repressed HIF‐1α expression. Consequently, N‐MYC gene 1, a downstream gene of HIF1a, is increased. Under this mechanism, SC144 sensitizes ovarian cancer cells to Olaparib, Carboplatin, and Cisplatin both in vitro and in vivo.^17^ Recently, Xu et al.^18^ demonstrated that IL‐6 upregulates HIF‐1α expression by inciting phosphorylation and nuclear localization of STAT3 under hypoxic conditions. However, it is not the only regulatory mechanism of HIF‐1α expression. Through the IL‐6/STAT3/HIF‐1α feedback loop, IL‐6 aggravates cisplatin resistance in ovarian cancer cells.

### 2.4 IL‐6 increased the expression of androgen receptor and estrogen receptor

Ovarian tissue exhibits specific physiological properties and endocrine functions, which contribute to its heightened sensitivity to hormone stimulation. Ovarian cancer is considered a hormone‐dependent cancer, as are breast cancer and prostate cancer. The concept of a hormonal etiology of epithelial ovarian cancer was proposed by HA Risch in 1998.^19^ Since this discovery, the literature has shown that the androgen receptor (AR) acts as a key driver of Paclitaxel resistance in ovarian cancer.^20^ As a ligand of Paclitaxel, Toll‐like receptor 4 (TLR4) enhances IL‐6 expression, and in turn, AKT phosphorylation capabilities level is markedly augmented by TLR4/IL‐6.^21^, ^22^ As demonstrated in in vitro ovarian cancer cell models, TLR4/IL‐6 provokes AR expression via the PI3K/AKT signaling pathway,^22^ among which phosphorylated AKT is essential for the expression of AR. Wang et al.^23^ demonstrated that IL‐6 activates ER signaling through MEK/ERK and PI3K/AKT pathways, leading to tamoxifen resistance in ovarian cancer cells.

### 2.5 IL‐6 induces paclitaxel resistance by modulating epithelial‐to‐mesenchymal transition

The tumor microenvironment involves tumor cells, fibroblasts, immune cells, muscle cells, nerves, and vessels. Cancer‐associated fibroblasts (CAFs) are the main source of IL‐6. In vitro studies using ovarian cancer cells and CAF‐conditioned media have demonstrated that CAFs reinforce Paclitaxel resistance by activating the epithelial‐to‐mesenchymal transition (EMT) through the IL‐6/Janus kinase 2 (JAK2)/STAT3 axis; these findings are further supported by clinical tissue analysis showing that elevated interstitial IL‐6 correlates with reduced chemotherapy sensitivity.^24^ EMT is associated with both drug resistance and cancer stem cells (CSCs) in several cancers.^25^ CSCs are found to be more drug‐resistant than non‐CSCs. EMT is an important node for cancer metastasis, which is tightly associated with CSCs.^25^, ^26^ However, the link between the two still requires further identification. The clarification of this relationship has the potential to uncover more treatment strategies in tumor therapy.

### 2.6 IL‐6 induced signaling pathways

**FIGURE 1:**
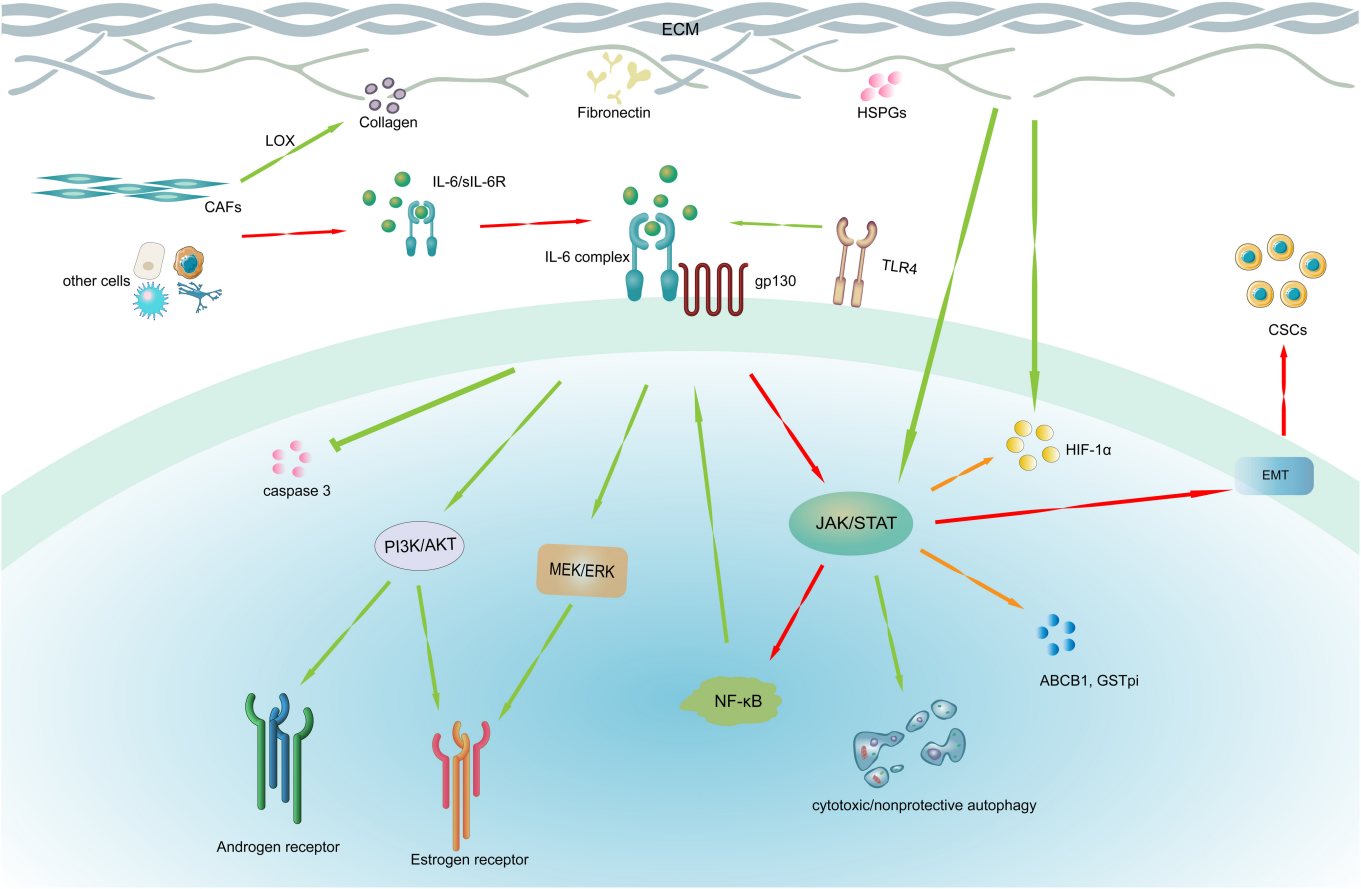
The role of IL‐6 in the ovarian cancer treatment resistance mechanism.

As a vital immunoregulatory cytokine, IL‐6 mediates chemoresistance through various signaling pathways (Figure 1), with the JAK2/STAT3 signaling pathway being particularly prominent in ovarian cancer.^27^ STAT3 induces the chemoresistance of ovarian cancer cells through multiple mechanisms. Together with IL‐6, it forms a positive feedback loop, as IL‐6 activates STAT3 and the phosphorylated STAT3 promotes IL‐6 transcription.^28^ Accumulating evidence has shown that phosphorylated STAT3 is highly expressed in Paclitaxel‐resistant and cisplatin‐resistant ovarian cancer cell lines.^29^, ^30^ Additionally, the IL‐6/STAT3 signaling pathway activates NF‐κB, which in turn promotes IL‐6 expression, forming another positive feedback loop.^31^

Finally, IL‐6 promotes the transcription of activating transcription factor 6 by activating STAT3 phosphorylation. Activating transcription factor 6 stimulates autophagy through endoplasmic reticulum stress, which assists tumor cells in producing resistance to Cisplatin and Paclitaxel. Devoted to degrading and recycling cellular components, autophagy is more complex in tumor progression. Indeed, autophagy remains a controversial issue so far, as it presents two contrasting features in cancer development. In the initial stage of ovarian cancer, autophagy inhibits tumor progression by phagocytizing DNA‐damaged cells, a mechanism referred to as “protective autophagy”. In contrast, once tumors reach the advanced stages, autophagy, driven by the tumor microenvironment, promotes the progression of the tumor by enabling cancer cells to survive in stressful or hypoxic environments and resist chemotherapeutic agents.^32^ In this context, autophagy works as “cytotoxic/nonprotective autophagy”, protecting ovarian cancer cells from chemotherapy and thus limiting its effectiveness.^33^ Further analysis is needed to determine the optimal application of autophagy‐targeting therapies in cancer treatment.

Moreover, in vitro studies have demonstrated that IL‐6 induces chemoresistance in ovarian cancer cells through Ras, Raf, MEK, or ERK‐related signaling pathways.^13^, ^34^ In addition to chemotherapy resistance, IL‐6 has also been implicated in resistance to anti‐VEGF antibody treatment in patients, likely through its proangiogenic effects mediated via similar pathways in both in vivo and ex vivo models.^34^

### 2.7 Oncostatin M

Oncostatin M (OSM), as an IL‐6 family member, is mainly secreted by immune cells such as T cells, monocytes, macrophages, neutrophils, and dendritic cells. OSM participates in key processes including, but not limited to, inflammation, hematopoiesis, CSCs maintenance, drug resistance, and metastatic progression.^35^ Compared to cells from other tumor microenvironments or normal ovarian tissue, ovarian cancer cells express higher levels of OSM receptor, and this differential expression has been observed in both patient‐derived tumor samples and established ovarian cancer cell lines.^36^ Upon binding to gp130 via OSM receptor β or LIFRβ, OSM influences the downstream signaling pathways including JAK/STAT, MAPK, and PI3K/AKT signaling pathways.^35^, ^37^ OSM induces epithelial‐mesenchymal plasticity by STAT3 phosphorylation and TGF‐β/SMAD signaling cooperation, as evidenced by the upregulation of EMT markers and changes in CD24/CD44 expression in vitro. Interestingly, CSC property maintenance requires persistent OSM exposure.^38^

## 3 IL‐11 IN OVARIAN CANCER

Under physiological conditions, IL‐11 is quite lowly expressed and hardly detectable. A low level of IL‐11 is responsible for platelet recovery, erythropoiesis, and bone cell proliferation and differentiation.^39^ Only when the inflammatory response or carcinogenesis occurs are IL‐11 levels notably raised. Strong evidence indicates IL‐11 is generally expressed in ovarian epithelial cells.^40^ Elevated IL‐11 is generally correlated to a higher tumor grade except for in the setting of bladder cancer.^41^ Nevertheless, unlike the proinflammatory responses incited by IL‐6, IL‐11 is considered an anti‐inflammatory cytokine.^42^ IL‐11 has also been implicated in several other aspects of tumor biology, including, but not limited to, stimulation of angiogenesis, hypoxic conditions, and drug‐resistance.^43^ In platinum‐resistant ovarian cancer cells, IL‐11 expression is intensified.^44^, ^45^ In platinum‐resistant ovarian cancer cells, elevated reactive oxygen species induce FOSL1 expression, which binds to the IL‐11 promoter and drives its autocrine upregulation. The resulting increase in IL‐11 activates JAK2/STAT5 signaling, and either pharmacologic inhibition of JAK2 with LY2784544 or neutralization of IL‐11 restores cisplatin sensitivity in both in vitro and in vivo models.^46^ Clinically, ovarian tumors from platinum‐resistant patients exhibit higher IL‐11 levels and increased JAK2 phosphorylation, correlating with shorter progression‐free and overall survival. Additionally, like IL‐6, secreted CAFs IL‐11 instigate chemoresistance through the gp130/JAK2/STAT3/Bcl2 signaling pathway in gastric cancer.^47^ Although demonstrated in gastric cancer models, this mechanism may be conserved in ovarian cancer and warrants further investigation. Targeting IL‐11‐mediated pathways is a promising strategy to overcome multidrug‐resistant cancers in clinical practice.

Lastly, LIF, another IL‐6 family factor, also plays an active part in the development of platinum resistance in ovarian cancer.

## 4 INTERLEUKIN‐8 IN OVARIAN CANCER

Interleukin‐8 (IL‐8), also known as CXCL8, is a proinflammatory CXC chemokine (or α‐chemokines) secreted by monocytes, tumor cells, endothelial cells, and neutrophils.^48^ IL‐8 mediates biological effects usually through binding to CXCR1 and CXCR2. IL‐8 is highly expressed in ovarian cancer, which might be influenced by inflammatory factors, androgens, estrogens, chemotherapy, and hypoxia.^49^, ^50^, ^51^ This observation is supported by immunohistochemical analysis of tumor tissues from ovarian cancer patients (*n* = 102), which demonstrated that elevated IL‐8 expression correlates with poor clinical outcomes. Correspondingly, functional studies using orthotopic xenograft models further confirmed that targeting IL‐8 improves sensitivity to docetaxel in both taxane‐sensitive and ‐resistant ovarian cancer cells.^49^ Autocrine production of IL‐8 by ovarian cancer cells promotes resistance to chemotherapy by reducing proteolytic activation of caspase‐3.^48^ Moreover, IL‐8 promotes ovarian cancer cell drug resistance by inducing the expression of MDR1, apoptosis inhibitor genes (*Bcl‐2*, *Bcl‐xL*, and *XIAP*), and by activating the Ras/MEK/ERK and PI3K/Akt signaling pathways.

## 5 FIBROBLAST GROWTH FACTOR

Fibroblast growth factor (FGF) plays a crucial role in regulating fundamental developmental pathways, such as angiogenesis, cell migration, and tissue injury repair. Therefore, it is easily hijacked by tumor cells to provide proper conditions for tumor development.^49^ In 2004, Loris De Cecco et al.^52^ claimed FGF2, secreted by epithelial cells, incites cell dispersal and morphological changes in epithelial morphology. Moreover, FGF2 prompts cell proliferation and invasion through neovascularization.^53^ By 2012, Smith et al.^54^ demonstrated for the first time that FGF1 is differentially expressed in aggressive high‐grade serous ovarian cancer, as determined by qRT–PCR analysis of 187 human ovarian tumor samples and correlated with tumor histology, chemotherapy response, and survival. In contrast with platinum‐sensitive cells, FGF1 expression is consistently higher in platinum‐resistant ovarian cancer cell lines. Accordingly, the high level of FGF1 predicts a worse overall survival.^54^, ^55^ Aberrant FGF and FGF receptor (FGFR) drive important signaling pathways related to tumor progression. Amplifications of FGFR subtypes, FGFR1 and FGFR2, are widely described in cancers.^56^, ^57^ These effects were demonstrated through FGFR1 and FGFR2 knockdown using shRNA in ovarian cancer cell lines, which resulted in reduced proliferation and enhanced cisplatin sensitivity in vitro. Moreover, FGFR2 inhibition significantly reduced tumor growth and enhanced cisplatin efficacy in xenograft models.^58^ Several inhibitors of FGFR have been applied in clinical trials and have promising results. Nevertheless, the appearance of acquired resistance leads to disease progression.^59^

## 6 GRANULOCYTE‐MACROPHAGE COLONY‐STIMULATING FACTOR IN OVARIAN CANCER

Granulocyte‐macrophage colony‐stimulating factor (GM‐CSF) is a glycoprotein produced by various cell types including lymphocytes, macrophages, fibroblasts, endothelial cells, chondrocytes, and tumor cells.^60^, ^61^ GM‐CSF/GM‐CSFR signaling acts as a key regulator of tumor‐associated macrophage differentiation, function, and survival.^62^ GM‐CSF interferes with the anti‐angiogenic therapeutic effect. In the ovarian cancer microenvironment, the major infiltrated cells include tumor‐suppressing CD8^+^ T lymphocytes and tumor‐promoting M2 macrophages.^63^, ^64^, ^65^ M2 macrophages are recruited to the intratumor of ovarian cancer in response to GM‐CSF signaling. M2 macrophages play a role in ovarian carcinogenesis, progression, and tumor relapse, with the function of fostering invasiveness, metastasis, and resistance to chemotherapy of ovarian cancer.^66^ In addition, downregulated MiR‐130b caused by hypermethylation exists in multidrug‐resistant ovarian cancer cells. Colony‐stimulating factor (CSF‐1) is a direct downstream target of miR‐130b, as confirmed by dual‐luciferase reporter assays and further validated by qRT‐PCR, immunohistochemistry, and ELISA in ovarian cancer tissues and cell lines. Both the knockdown of CSF‐1 or reactivation of miR‐130b expression using the DNMT inhibitor 5‐aza‐CdR were shown to sensitize drug‐resistant ovarian cancer cells to anticancer drugs.^67^

## 7 TRANSFORMING GROWTH FACTOR‐β IN OVARIAN CANCER

Transforming Growth Factor‐β (TGF‐β) is a widely criticized factor in multiple biological functions as it stimulates multiple signaling pathways of tumorigenesis and metastasis. Like CSF, TGF‐β also plays a dual role in tumor development. Initially, it acts as a suppressor to reduce tumor cell proliferation. However, as the tumor progresses, TGF‐β instigates EMT and fibrosis instead.^68^ In advanced‐stage high‐grade serous ovarian cancer, TGF‐β signaling is upregulated and is accompanied by global repression of miRNA expression, as observed through transcriptomic and miRNA expression analyses of patient tumor samples. Mechanistically, in vitro studies in ovarian cancer cell lines demonstrated that this effect is mediated by the non‐coding RNA nc886, which binds to Dicer and impairs its ability to process miRNAs.^69^ TGF‐β1 simultaneously activates SMAD2 and SMAD3 to form heterohexameric complexes to directly target genes involved in cellular processes, such as induction of chemoresistance.^70^ Collagen matrix in 3D culture impels EMT by activating TGF‐β/SMAD and Wnt/β‐catenin‐related pathways and upregulating the expression of Snail and Slug for tumor cell invasion and chemotherapy resistance.^71^ Consistent with the above results, the aggressiveness of ovarian cancer cells positively correlates with their resistance to chemotherapeutic drugs, such as Carboplatin, Doxorubicin, and Paclitaxel. In such cells, Slug maintains a high expression level. Slug alleviates the anticancer drug cytotoxicity by encouraging the phosphorylation of c‐Met. The resistance to doxorubicin is relieved when cells are treated with a c‐Met inhibitor.^72^ The TGF‐β1/AKT signaling pathway also plays a crucial role in the process of EMT. Zhang et al.^73^ reveal that overexpressed TGF‐β and EGFR boost EMT progression, CSC transformation, and multidrug resistance phenotype. These effects were observed in ovarian cancer cell lines treated with CD163^+^ TAM‐derived exosomes and were supported by immunohistochemical analysis of patient tumor tissues. Furthermore, with the TGFβ1/ERK/PHD2 axis, platinum resistance can form under HIF1a regulation.^74^ Therefore, inhibiting TGF‐β is a potential strategy for interrupting ovarian cancer invasion and metastasis.

## 8 TUMOR NECROSIS FACTOR

Tumor necrosis factor (TNF) is required in inflammation and apoptosis in the ovarian cancer microenvironment. TNF‐α is produced by multiple cell types, mainly by monocytes and neutrophil lineages.^75^

TNFα receptor expression is crucial for biological function executions of TNFα. TNFR1 is situated on most cells, whereas TNFRII is expressed only on endothelial cells, fibroblasts, and subsets of neurons and immune cells.^76^ Endogenous TNF implements tumor cell necrosis activity, causing tumor‐related inflammation.^77^ In ovarian cancer, TNF constitutes a malignant cell‐autonomous inflammatory cytokine network, contributing to tumor cell proliferation, invasion, and drug resistance, particularly through the interactions between tumor cells and macrophages. This autocrine inflammatory cytokine network was characterized in ovarian cancer cell and macrophage co‐culture models and further validated in vivo using TNF‐α knockout mice and antibody blockade approaches.^77^, ^78^ TNF‐α deficient mice are more immunized to skin cancer cells, which provides evidence to support that TNF exerts a positive driving force on tumorigenesis.^79^ TGF inhibitors or neutralizing antibodies have demonstrated potential in improving patient survival. Besides, the TGF inhibitors enhance the efficacy of chemotherapy, making them promising therapeutic agents. TNF‐α induces drug‐resistant status in multiple types of cancers to prolong the survival of tumor cells exposed to Cisplatin. In comparison with IL‐6, TNF‐α activates NF‐κB at a much higher level.

In addition, TNF‐α/NF‐κB induces the activation of Tumor necrosis factor α‐induced protein 8 (TNFAIP8).^80^, ^81^ As an oncogenic and anti‐apoptotic protein, TNFAIP8 gives impetus to cell survival and inhibits the activity of apoptotic proteins, caspase 3 and caspase 8. TNFAIP8 is overexpressed in various cancer types and contributes to poor clinical prognosi.^82^, ^83^ Additionally, TNFAIP8 expression is markedly increased in platinum‐resistant tumors versus normal ovarian tissue, indicating that TNFAIP8 is associated with ovarian cancer drug resistance. OVCAR‐3 cells with downregulated TNFAIP8 exhibit higher chemosensitivity and increased expression levels of autophagy‐related proteins (Beclin 1 and LC II).^84^

Furthermore, TNF‐α increases the level of phosphorylated ERK and phosphorylated p38 MAPK in OC cells.^85^

## 9 VASCULAR ENDOTHELIAL GROWTH FACTOR IN OVARIAN CANCER

The vascular endothelial growth factor (VEGF) family comprises multiple ligands, including VEGFA, VEGFB, VEGFC, VEGFD, VEGFE, placenta growth factor 1, and 2. These ligands exert their effects through three receptors: VEGFR type 1, 2, and 3. The physiological mechanism of the ovary is wired with abundant blood vessels to provide nutrients.^86^ The VEGF/VEGFR signaling pathway is widely recognized for stimulating angiogenesis. A high VEGFA expression level is associated with advanced stages of malignancy, malignant ascites formation, and acquisition of an invasive phenotype. Extensive evidence indicates VEGF/VEGFR promotes ovarian cancer progression and correlates with poor prognosis.^87^, ^88^ However, the emergence of anti‐angiogenic therapy targeting VEGF/VEGFR has achieved gratifying curative effects in several solid tumors, including recurrent ovarian cancer.^89^ Bevacizumab, a VEGF monoclonal antibody, has shown a clinical benefit, even for cisplatin‐resistant recurrent ovarian cancer.^90^ Besides, Bevacizumab in combination with Atezolizumab is a feasible treatment strategy for advanced recurrent epithelial ovarian cancer patients who may have a cisplatin‐resistant status.^91^ However, resistance to VEGF/VEGFR‐targeting drugs has been reported, which may result from hypoxia‐induced signaling and suppression of antitumor immunity, as suggested by analyses of immune cell populations, hypoxic conditions, and GM‐CSF‐regulated MDSC function in preclinical murine tumor models and clinical samples.^92^

## 10 INTERLEUKIN‐4 IN OVARIAN CANCER

Interleukin‐4 (IL‐4), primarily secreted by ovarian cancer cells, promotes resistance to anti‐PD‐1 immunotherapy by shaping an immunosuppressive tumor microenvironment (TME) through macrophage modulation. This conclusion is based on a spatial functional genomics screen (Perturb‐map) performed in preclinical ovarian cancer models, which recapitulated tumor heterogeneity and revealed that IL‐4 directs localized TME composition, driving clonal selection and immunotherapy resistance. These findings highlight IL‐4 signaling as a promising therapeutic target.^93^

## 11 MONOCYTE CHEMOATTRACTANT PROTEIN‐1 IN OVARIAN CANCER

Monocyte chemoattractant protein‐1 (MCP‐1), a key chemokine involved in monocyte recruitment and tumor‐promoting inflammation, has been found to be associated with a higher recurrence risk in epithelial ovarian cancer patients undergoing cytoreduction combined with hyperthermic intraperitoneal chemotherapy. This conclusion is drawn from a clinical cohort study analyzing serum cytokine levels in 34 epithelial ovarian cancer patients before and after cytoreduction combined with hyperthermic intraperitoneal chemotherapy, with follow‐up assessing progression‐free survival. Elevated baseline and post‐treatment MCP‐1 levels, as well as greater changes over time, predicted shorter progression‐free survival. Together with IL‐8 and IL‐13, MCP‐1 may contribute to a postoperative inflammatory cascade that facilitates chemoresistance or early recurrence.^94^

## 12 INTERFERON‐ε IN OVARIAN CANCER

Interferon‐ε (IFNε), an intrinsic tumor suppressor lost during ovarian cancer development, exerts anti‐tumor effects by directly targeting tumor cells and activating anti‐tumor immunity. In preclinical models, IFNε enhances T cell and NK cell responses while limiting immunosuppressive cells, suggesting its loss may contribute to immune evasion and therapeutic resistance in advanced ovarian cancer.^95^

## 13 ANTI‐INFLAMMATORY PHYTOCHEMICALS—CHEMOTHERAPY SENSITIZERS

Anti‐inflammatory phytochemicals are derived from active extracts of natural products and have beneficial properties including greater spatial complexity, fewer heavy atoms, and fewer side effects. Therefore, natural compounds and their derivatives are attractive in assisting with overwhelming the drug resistance of chemotherapy. Based on numerous published in vitro and in vivo data, assorted anti‐inflammatory phytochemicals contribute to the regulation of the tumor microenvironment. Several of them have been shown to act on drug‐resistant ovarian cancer cells by enhancing apoptosis‐related pathways and inhibiting the secretion of proinflammatory cytokines. As chemotherapy sensitizers, phytochemicals improve the therapeutic response when combined with clinical first‐line drugs. We introduce the phytochemicals related to the chemoresistance of ovarian cancer as follows (Table 1).^17^, ^46^, ^96^, ^97^, ^98^, ^99^, ^100^, ^101^, ^102^

**TABLE 1:** The function of cytokines inhibitors or antibodies in chemotherapy resistance in ovarian cancer.

Drugs	Mechanism	Related cytokines	Sensitive	Cell lines	References	Manner
Siltuximab	The monoclonal antibody with high binding affinity for IL‐6	IL‐6	Platinum	IGROV‐1/TOV21G/TOV112D/SKOV‐3	96	Dose–time‐dependent
SC144	Binding to gp130, induces gp130 phosphorylation and inhibits the expression of downstream target genes of the gp130 pathway	IL‐6 complex	Olaparib/carboplatin/cisplatin	OVCAR‐8/SK‐OV‐3/LN‐Cap	17	Dose–time‐dependent
ABT‐737	The inhibitor of Bcl‐2	Bcl‐2	Cisplatin	SKOV‐3/OVCAR‐3	97	Dose–time‐dependent
Stattic	The inhibitor of STAT3, reductions in the expression of the anti‐apoptosis protein and phosphorylated‐Akt levels	STAT3	Cisplatin	OV2008/C13	98	Dose–time‐dependent
LY2784544	The inhibitor of JAK2	JAK2	Platinum	SKOV3/IGROV1	46	Dose–time‐dependent
AZD4547 and SU5402	The inhibitor of FGFR	FGFR	Cisplatin/carboplatin	A2780/cisplatin‐resistant‐A2780/SK‐OV‐3/CaOV3	99	Dose‐dependent
Tocilizumab	Antibody against IL‐6R and inhibits IL‐6R signaling pathway	IL‐6R/sIL‐6R	Cisplatin/carboplatin/doxorubicin	JHOC‐5/JHOC‐7/JHOC‐8/JHOC‐9/HAC‐2/RMG‐I/RMG‐II/OVTOKO/OVMANA/OVISE/ES‐2	100	Dose‐dependent
Bazedoxifene	Inhibition of GP130/STAT3 and a selective estrogen modulator	GP130/STAT3/ER	Paclitaxel	A2780/OVCA433/SKOV3/TOV112D	101	Dose‐dependent
AG490	JAK2 kinase inhibitor and inhibits the expression of MDR1	JAK2	Paclitaxel	OC3/TAX300	102	Dose‐dependent

As stated, IL‐6/STAT3 plays a vital role in the development of drug‐resistant ovarian cancer. CDDO‐Me, a synthetic triterpenoid, hinders the nuclear translocation and phosphorylation of STAT3 by diminishing the secretion of IL‐6 and then decreasing the phosphorylation of JAK2 and Src. This has been demonstrated in multidrug resistant ovarian cancer cell lines to partially reverse ovarian cancer drug‐resistant phenotypes.^103^ Moreover, CDDO‐Me can inhibit NF‐κB activation, which in turn promotes IL‐6 secretion.^104^, ^105^

Genistein, a soy isoflavone, has been reported in ovarian cancer cell lines to induce autophagic cell death by arresting the cell cycle at the G2/M phase,^106^, ^107^ and exerts a chemosensitizing effect when combined with chemotherapy. Genistein activates the NF‐κB signaling pathway, leading to the downregulation of Bcl‐2, Bcl‐xL, survivin, and c‐IAP2.^108^ Meanwhile, Genistein alters drug resistance by regulating the PI3K/AKT/mTOR, NRF2 signaling pathways, and the TLR4‐androgen receptor axis. This evidence suggests that Genistein is a promising synergistic drug in ovarian cancer combination therapy.^108^, ^109^

Limonin is extracted from *Evodia rutaecarpa*. It intensifies caspase‐3 activity through activation of p53 to induce cell apoptosis and reverse drug resistance in cisplatin‐resistant ovarian cancer cells.^110^ Evodiamine, another component of *E. rutaecarpa*, reduces ovarian cancer chemoresistance by repressing P‐glycoprotein expression and CDC2/ERK phosphorylation.

Curcumin is an active component from *Curcuma longa*. Curcumin can not only prevent ovarian carcinogenesis but can also influence the sensitization of chemotherapy and antitumor drugs. Curcumin has been demonstrated in ovarian cancer cell lines and murine models to influence chemosensitization, hindering NF‐κB, Wnt/β‐catenin, AKT/mTOR/p70S6K pathways and reducing the expression of P‐glycoprotein to restore ovarian cancer drug sensitivity.^111^, ^112^ The combination of Curcumin and Resveratrol significantly impedes the PI3K/AKT/mTOR pathway, thereby sensitizing ovarian cancer cells exposed to Cisplatin.^113^

Resveratrol is a phytoalexin found in grapes. Resveratrol reverses ovarian cancer doxorubicin‐resistant status by suppressing *MDR1*, *MRP1*, and *Bcl‐2* gene expression levels. Meanwhile, Resveratrol is also capable of saturating ovarian cancer stem cells with CD133+ signatures. MDR1 is confirmed as a downstream target gene of the Hedgehog pathway, which promotes the resistance of ovarian cancer cells to chemotherapeutic drugs by multiple means.^114^ Trans−4,4′‐dihydroxy stilbene derivatives from resveratrol arrest the cell cycle in the S phase by inducing the degradation of RRM2, inhibiting DNA damage repair response to overcome cisplatin resistance in ovarian cancer cell model.^115^

Triptolide has been demonstrated in ovarian cancer cell lines and animal models to reduce ovarian cancer chemoresistance by interrupting PI3K/AKT/GSK3‐β and JAK/STAT3 signaling pathways.^116^, ^117^ Apigenin counteracts chemoresistance via downregulating the phosphorylation of Akt and the expression of TAM RTKs and Mcl‑1 in ovarian cancer cells.^118^

Except for the characterizations noted above, Silibinin has been shown in cisplatin‐ and paclitaxel‐resistant ovarian cancer cell lines to significantly restore drug sensitivity by inhibiting proliferation, inducing apoptosis, and reducing cell‐matrix adhesion. While its direct antitumor effect on resistant cells is limited, Silibinin enhances the efficacy of cisplatin and paclitaxel. Moreover, it mitigates chemotherapy‐induced hepatotoxicity in LO2 cells by protecting against DNA damage and promoting cell survival.^119^ Moreover, Silibinin effectively resumes doxorubicin sensitivity via targeting GLUT, P‐glycoprotein, as well as MDR genes.^120^, ^121^

Despite the promising chemosensitizing and anti‐inflammatory potential of natural compounds such as Curcumin, Limonin, and Resveratrol, their application remains limited. To date, supporting evidence is largely restricted to in vitro studies, with a lack of validation in preclinical models or clinical trials. These compounds are not yet used in clinical practice, and no patient‐based studies confirm their efficacy in enhancing treatment outcomes for ovarian cancer. Moreover, phytochemicals have not been systematically characterized or categorized, and efforts are needed to identify shared structural features and common molecular targets. Substantial research is still required before natural ingredients can be integrated into standardized adjuvant cancer therapies.

## 14 ECM–CYTOKINE CROSSTALK IN OVARIAN CANCER DRUG RESISTANCE

A growing body of evidence suggests that extracellular matrix (ECM) components, particularly collagen and fibronectin, are not merely passive structural barriers but actively shape the cytokine landscape within the tumor microenvironment of ovarian cancer. In epithelial ovarian cancer, excessive collagen deposition and remodeling have been directly associated with impaired drug penetration and resistance to platinum‐based chemotherapy, such as cisplatin.^122^ One of the key drivers of ECM remodeling is the lysyl oxidase (LOX) family of enzymes, which are highly expressed by CAFs. LOX‐mediated cross‐linking of collagen fibers significantly increases ECM stiffness, which is sensed by integrin β1 on tumor and stromal cells.^123^ The engagement of integrin β1 subsequently activates downstream mechanotransduction pathways, including focal adhesion kinase (FAK), Src, and the Rho/ROCK axis.^123^ These pathways collectively promote the transcription and secretion of pro‐inflammatory and pro‐survival cytokines such as IL‐6 and IL‐8, thereby reinforcing chemoresistance.^124^

This bidirectional matrix–cytokine interplay creates a pro‐tumorigenic mechanobiochemical niche that supports cancer cell survival, stemness, and therapy resistance. Disrupting this circuit has shown therapeutic potential: inhibition of LOX activity using beta‐aminopropionitrile has been reported to reduce matrix stiffness and IL‐6 levels, improving drug delivery and restoring chemosensitivity in preclinical models.^125^ Similarly, pharmacological blockade of the integrin–FAK/Src axis attenuates cytokine secretion and tumor‐promoting signals downstream of ECM mechanotransduction, highlighting a promising strategy to overcome ECM‐driven drug resistance.^124^

## 15 TME HETEROGENEITY: HYPOXIA‐DRIVEN CAF ACTIVATION AND METABOLIC REWIRING IN OVARIAN CANCER RESISTANCE

In ovarian cancer, hypoxia leads to the activation of HIF1α in both tumor cells and CAFs, which triggers the secretion of profibrotic and proangiogenic cytokines such as TGFβ and VEGF, thereby promoting stromal remodeling and neovascularization.^126^

Through the TGFβ signaling axis, tumor‐derived factors—including lysophosphatidic acid and exosomes—can induce the differentiation of mesenchymal stem cells into CAFs. These activated CAFs, in turn, upregulate the expression of IL6, CXCL12, and VEGFA in response to cancer‐derived TGFβ stimulation; ultimately enhancing metastasis and contributing to therapy resistance.^127^

In parallel, hypoxia‐driven metabolic rewiring involves increased expression of ceramide transport proteins and sphingosine kinase 1 and 2 isoenzymes, which are essential regulators of sphingolipid biosynthesis. Elevated levels of these enzymes have been correlated with resistance to chemotherapeutic agents, including cisplatin, paclitaxel, and tamoxifen in ovarian cancer.^128^

## 16 CONCLUSION

Cytokines play crucial roles in tumor progression, relapse, and drug resistance of ovarian cancer. Other chemokines may also potentially contribute to drug resistance in direct or indirect ways. For example, chemokine CXCL2 maintains cancer cell stemness and activates the ATR/CHK1 signaling pathway to promote platinum resistance in ovarian cancer.^129^ Additionally, CCL20 promotes ovarian cancer resistance by modulating ABCB1 expression and Notch1 signaling pathway.^130^ Related pathways such as Wnt/β‐catenin, STAT3, and PI3K/Akt are also involved in modulating ovarian cancer drug sensitivity status.^131^, ^132^

Many patients can eventually develop resistance to this single‐target therapy and experience disease progression. Accordingly, Table 2 summarizes the inhibitors or antibodies targeting various cytokines.^103^, ^108^, ^110^, ^111^, ^116^, ^118^, ^120^, ^133^, ^134^, ^135^ As pleiotropic mediators, these cytokines can activate multiple pathways to promote tumor progression and help cancer cells adapt to extremely complex and heterogeneous living environments. Given the multifaceted roles of cytokines, natural plant ingredients have gradually drawn attention for their therapeutic potential. Indeed, many active phytochemicals can target multiple signaling pathways to conquer drug resistance.

**TABLE 2:** Major mechanisms of natural compounds in drug‐resistance in ovarian cancer.

Compound name	Pathways/targets	Related cytokines	Sensitization target	Cell line	References	Manner
CDDO‐Me	IL6/JAK/Src/STAT3; OSM/STAT3	IL‐6; OSM	Paclitaxel; Cisplatin	Cisplatin‐resistant‐SKOV3/A2780/OVCAR8	103	Dose–time‐dependent
Limonin	P53/Caspase‐3		Cisplatin	Cisplatin‐resistant‐SKOV3/A2780/RMUG‐S	110	Dose‐dependent
Evodiamine	RAS/MAPK		Paclitaxel	Paclitaxel‐resistant‐A2780	116	Dose–time‐dependent
Resveratrol	HG/BMI‐1/MDR‐1; JAK/STAT; PI3K/AKT/GSK3‐β/mTOR; caspase‐3	IL‐6	Platinum	SKOV3	133	Dose–time‐dependent
Triptolide	PI3K/AKT/GSK3‐β; JAK/STAT3	IL‐2/TNF‐α	Platinum	Cisplatin‐resistant‐COC1/SKOV3/DDP cells	134	Dose–time‐dependent
Apigenin	AKT;TAM RTKs (tyro3, axl, mer); Mcl1		Paclitaxel; Cisplatin	Taxol‐resistant‐SKOV3/SKOV3/DDP	118	Dose‐dependent
Silybin B	p‐gp; MDR; glut	IL‐6/TNF‐α/IL‐1α/COX‐2	Adriamycin; doxorubicin	Multidrug resistant ovarian sub‐line resistant to doxorubicin/A2780‐DOX	120	Dose‐dependent
Cardamonin	NF‐κB; mTOR		Paclitaxel; Cisplatin	SKOV3/PDC/taxol‐resistant‐SKOV3	135	Dose‐dependent
Genistein	NF‐κB; PI3K/AKT/mTOR; Nrf2; TLR4‐androgen receptor axis		Cisplatin, paclitaxel	SKOV3	108	Dose‐dependent
Curcumin	NF‐κB; phosphorylated STAT3; COX‐2; IL‐8; MMP‐9; VEGF; Wnt/β‐catenin; AKT/mTOR/p70S6K	IL‐8/STAT3/COX2/VEGF	Paclitaxel	SKOV3/SKOV3ip1/HeyA8/HeyA8‐MDR/A2780	111	Dose‐dependent

**FIGURE 2:**
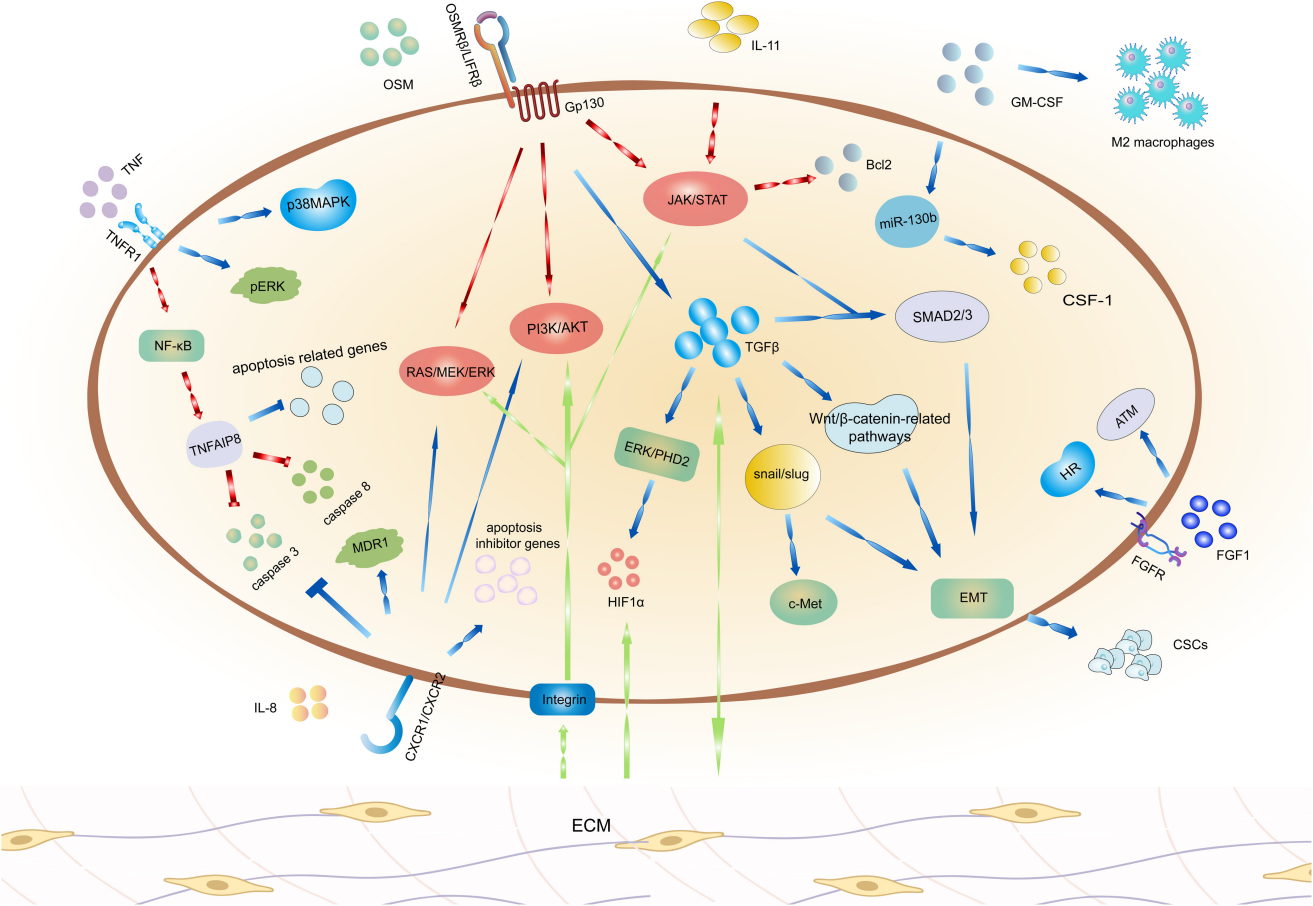
Other cytokines' role (OSM, IL‐11, IL‐8, FGF, GM‐CSF, TGF‐β, TNF) in ovarian cancer's therapeutic resistance mechanism.

In summary, the mechanisms of tumor drug resistance are intricate. Figure 2 depicts a quick overview of the endocrine signaling process, illustrating how cytokines, excluding IL‐6, may play a role in treatment resistance in ovarian cancer patients. The tumor microenvironment plays a key role in this process as it provides convenient conditions for the tumor cells to survive and expand, even in unfavorable circumstances. As one vital influencing point of tumor cells, the mechanism of cytokines in drug resistance is complex and individually differentiated, thus requiring personalized targeted therapy. At the same time, it is necessary to integrate databases to find commonalities to escort the treatment. When evaluating patients receiving chemotherapy, single inhibition cannot block the influence of cytokines on the drug resistance mechanism, so the emergence of multi‐targeted drugs is necessary. Further research is needed to evaluate the mechanism of drug resistance to expand our knowledge on how to maximize patient beneficence. Emerging natural pharmaceutical ingredients sensitize chemotherapy in clinics, which can contribute to the need to overcome drug resistance and promote quality of life. However, deep analysis and integration of drug structure and pharmacokinetics are needed to provide more basic application suggestions for healthcare centers. Achieving long‐term goals such as sensitizing patients to chemotherapy, prolonging overall survival, and improving quality of life requires the integration of diverse resources.

## AUTHOR CONTRIBUTIONS

**Lu Wang:** Writing – original draft; writing – review and editing; resources; visualization. **Yunxia Zang:** Writing – original draft; data curation; validation; visualization. **Rebeka Dejenie:** Writing – review and editing; writing – original draft. **Junyuan Bing:** Data curation; visualization; investigation; formal analysis. **Shixia Dong:** Writing – original draft; software; visualization. **Yanfei Zhang:** Writing – original draft; resources; validation. **Yi Zhu:** Conceptualization; funding acquisition; writing – review and editing; project administration; supervision. **Jingjing Li:** Writing – review and editing; conceptualization; supervision; funding acquisition; project administration.

## CONFLICT OF INTEREST STATEMENT

The authors declare no conflicts of interest.
